# Potent neutralizing antibodies in humans infected with zoonotic simian foamy viruses target conserved epitopes located in the dimorphic domain of the surface envelope protein

**DOI:** 10.1371/journal.ppat.1007293

**Published:** 2018-10-08

**Authors:** Caroline Lambert, Mathilde Couteaudier, Julie Gouzil, Léa Richard, Thomas Montange, Edouard Betsem, Réjane Rua, Joelle Tobaly-Tapiero, Dirk Lindemann, Richard Njouom, Augustin Mouinga-Ondémé, Antoine Gessain, Florence Buseyne

**Affiliations:** 1 Unité d’Épidémiologie et Physiopathologie des Virus Oncogènes, Institut Pasteur, Paris, France; 2 UMR CNRS 3569, Institut Pasteur, Paris, France; 3 Sorbonne Paris Cité, Cellule Pasteur, Université Paris Diderot, Institut Pasteur, Paris, France; 4 University of Yaounde I, Yaounde, Cameroon; 5 CNRS UMR 7212, INSERM U944, Institut Universitaire d’Hématologie, Hôpital Saint-Louis, Université Paris Diderot, Sorbonne Paris Cité, Paris, France; 6 Institute of Virology, Medical Faculty “Carl Gustav Carus”, Technische Universität Dresden, Dresden, Germany; 7 Center for Regenerative Therapies Dresden, Technische Universität Dresden, Dresden, Germany; 8 Laboratoire de Virologie, Centre Pasteur du Cameroun, Yaoundé, Cameroon; 9 Unité de Rétrovirologie, Centre International de Recherche Médicale de Franceville, Franceville, Gabon; King's College London, UNITED KINGDOM

## Abstract

Human diseases of zoonotic origin are a major public health problem. Simian foamy viruses (SFVs) are complex retroviruses which are currently spilling over to humans. Replication-competent SFVs persist over the lifetime of their human hosts, without spreading to secondary hosts, suggesting the presence of efficient immune control. Accordingly, we aimed to perform an in-depth characterization of neutralizing antibodies raised by humans infected with a zoonotic SFV. We quantified the neutralizing capacity of plasma samples from 58 SFV-infected hunters against primary zoonotic gorilla and chimpanzee SFV strains, and laboratory-adapted chimpanzee SFV. The genotype of the strain infecting each hunter was identified by direct sequencing of the *env* gene amplified from the buffy coat with genotype-specific primers. Foamy virus vector particles (FVV) enveloped by wild-type and chimeric gorilla SFV were used to map the envelope region targeted by antibodies. Here, we showed high titers of neutralizing antibodies in the plasma of most SFV-infected individuals. Neutralizing antibodies target the dimorphic portion of the envelope protein surface domain. Epitopes recognized by neutralizing antibodies have been conserved during the cospeciation of SFV with their nonhuman primate host. Greater neutralization breadth in plasma samples of SFV-infected humans was statistically associated with smaller SFV-related hematological changes. The neutralization patterns provide evidence for persistent expression of viral proteins and a high prevalence of coinfection. In conclusion, neutralizing antibodies raised against zoonotic SFV target immunodominant and conserved epitopes located in the receptor binding domain. These properties support their potential role in restricting the spread of SFV in the human population.

## Author summary

Foamy viruses are the oldest known retroviruses and have been mostly described to be nonpathogenic in their natural animal hosts. Simian foamy viruses (SFVs) can be transmitted to humans, in whom they establish persistent infection, as have the simian lenti- and deltaviruses that led to the emergence of two major human pathogens, human immunodeficiency virus type 1 (HIV-1) and human T lymphotropic virus type 1 (HTLV-1). Such cross-species transmission of SFV is ongoing in many parts of the world where humans have contact with nonhuman primates. We present the first comprehensive study of neutralizing antibodies in SFV-infected humans. We showed high titers of neutralizing antibodies in the plasma of most SFV-infected individuals. Neutralizing antibodies target the dimorphic portion of the envelope protein surface domain that overlap with the receptor binding domain. SFV-specific antibodies target epitopes conserved over 8 million years of co-speciation with their nonhuman primate host. Greater neutralization potency in infected individuals was statistically associated with smaller SFV-related hematological changes. In conclusion, our results suggest the protective action of neutralizing antibodies against SFV infection and spread in the human population.

## Introduction

Simian foamy viruses (SFVs) are complex retroviruses that are widely prevalent in nonhuman primates (NHPs) [1]. In animals, SFV replicate in the superficial cell layers of the buccal cavity [2] and are mostly transmitted through bites and licking [3]. Humans are not natural hosts of SFV, but can be persistently infected over several decades after a cross-species transmission event [4–7]. Most SFV-infected people were bitten by a NHP and are thus the first hosts of a zoonotic virus [6, 8]. Human infection with zoonotic SFV is thus a natural model to study the key steps of the emergence of retroviruses. Several NHP species live in the tropical forests of Central Africa, and people from rural areas are frequently exposed to their body fluids through hunting, butchering, and meat consumption. Several new zoonotic agents have emerged from simian reservoirs populating this region, including human immunodeficiency virus-1 (HIV-1), human T-cell leukemia virus-1 (HTLV-1), and Ebola and Monkeypox viruses [9]. We have established that the prevalence rates of SFV in South Cameroon are approximately 0.3% of the general population and greater than 20% for people who have been bitten by a NHP [8, 10, 11]. Our work and other studies on people infected with SFV from African NHP species have consistently reported the persistence of replication-competent virus and the presence of SFV DNA in blood cells [12–18]. Blood gorilla SFV DNA loads vary between 1 and 1000 copies/10^5^ cells [8, 18]. This is the range observed for blood HIV-1 DNA levels in HIV-1 infected humans [19].

From the immunological point of view, human SFV infection corresponds to the efficient control of a zoonotic retrovirus: SFVs persist throughout the lifetime of the host, but no major clinical impact for the infected host nor transmission to other human hosts has yet been described [4–6, 8]. SFVs show broad organ tropism in NHPs [2, 20]. *In vitro*, they are highly cytopathic for most cell lines [21]. In macaques, neutralizing antibodies prevent SFV transmission through blood transfusion [22]. SFV induces type I interferon (IFN) production in *in vitro* infected cells [23], and are susceptible to type I and type II IFNs and several restriction factors [7, 24–28]. Concerning immune responses, the only studies on human samples have shown SFV genome editing by apolipoprotein B mRNA-editing catalytic polypeptide (APOBEC) cytidine deaminases [29, 30] and the presence of neutralizing antibodies in one worker infected with a *chlorocebus* SFV [15].

SFV-specific antibodies induced in response to a zoonotic infection probably contribute to the restriction of viral replication in the infected hosts and the inhibition of human-to-human SFV transmission, two key steps in pathogen emergence. The antiviral function and properties of SFV-specific antibodies have yet to be described in humans. Accordingly, we aimed to perform an in-depth characterization of neutralizing antibodies raised by humans infected with a zoonotic SFV and their relationship with viral genotypes. We focused on gorilla SFV because these strains are found in approximately 70% of infected individuals in Cameroon and Gabon [8, 10, 31]. Furthermore, gorillas are phylogenetically close to humans. We quantified the neutralizing capacity of plasma samples from 58 SFV-infected hunters against primary zoonotic SFV strains [12]. We then studied the relationship between the neutralization specificity of plasma samples and the genotype of the strain infecting each hunter, defined the region of the SFV envelope protein (Env) targeted by the neutralizing antibodies, characterized the cross-recognition of various SFV genera, and investigated whether neutralization was associated with the characteristics of the SFV infection [32].

## Results

### Plasma from gorilla SFV-infected people efficiently neutralizes primary zoonotic SFV strains

**Fig 1:**
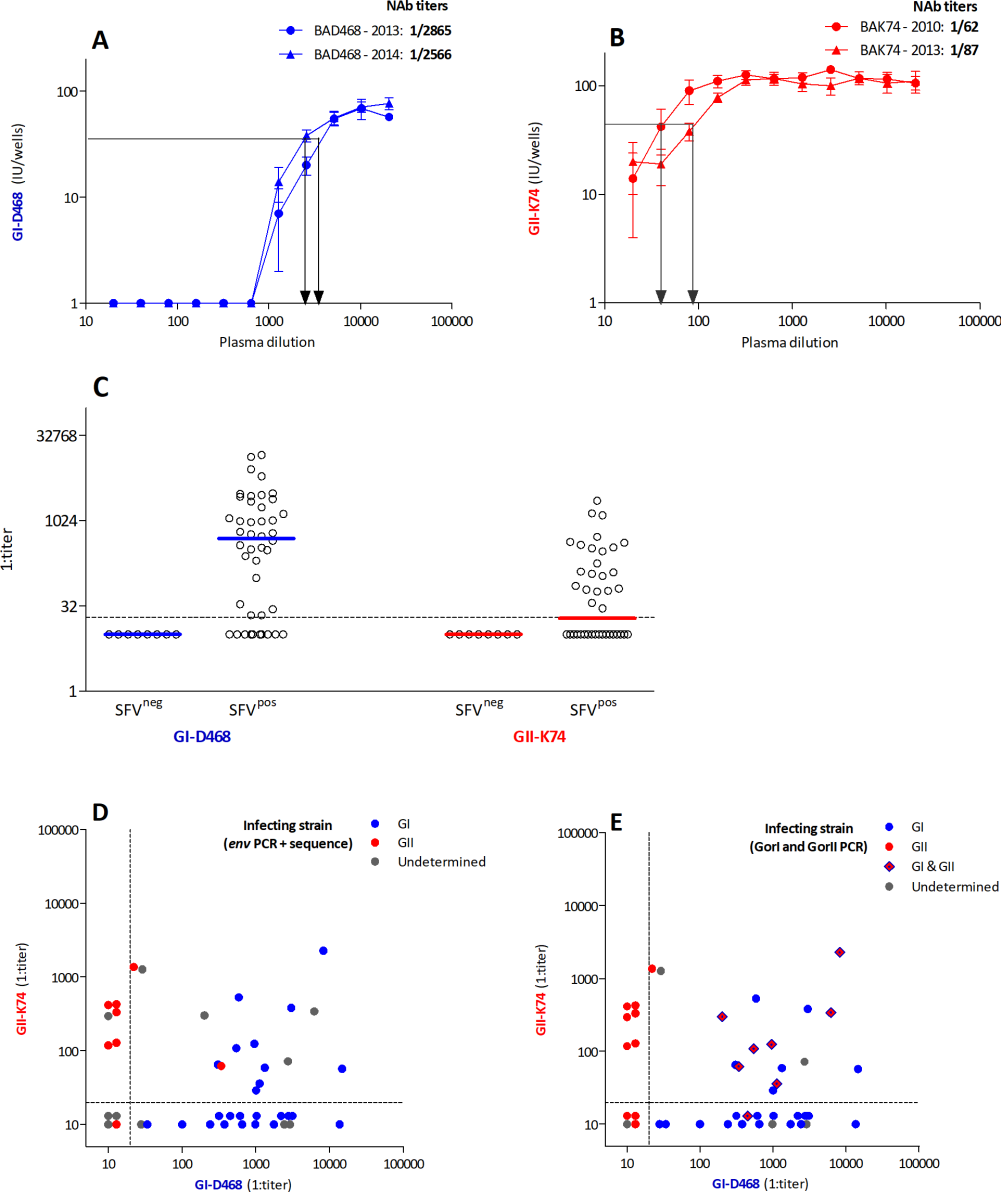
Neutralization of primary gorilla SFV strains by human plasma samples. Neutralization assays were carried out by infecting the GFAB indicator cells with SFV strains previously incubated with serial dilutions of human plasma samples. GI-D468 (A, blue) and GII-K74 (B, red) strains were incubated with autologous samples collected at two time points. The number of infected units per well (mean, SD) is presented as a function of plasma dilution. Arrows indicate neutralization titers. C. GI-D468 and GII-K74 strains were incubated with serial dilutions of plasma samples from 44 people infected with gorilla SFV (SFV^pos^) and eight uninfected controls (SFV^neg^). The neutralization titers against both strains are shown and the bars indicate the median values. D and E: The neutralization titers against GI-D468 (x axis) and GII-K74 (y axis) are presented for the SFV-infected individuals, with symbols colored according to their genotype. Panel D shows the genotypes previously obtained by PCR amplification of the *env* gene followed by sequencing [33]. Panel E shows genotypes defined by GI and GII-specific PCR described in this study. The dashed lines indicate the neutralization detection thresholds (1:20).

The primary strains isolated from our study population, representative of the two genotypes, were the tool of choice for the initial evaluation of neutralizing antibodies in the absence of data on the neutralization of gorilla SFV [12, 33]. The primary zoonotic GI-D468 and GII-K74 strains were neutralized by autologous plasma collected at two time points. Neutralization titers were very high (> 1:2,000) for plasma samples of BAD468 and moderate (< 1:200) for those of individual BAK74 (Fig 1A and 1B).

We quantified the neutralizing activity of plasma samples from 44 gorilla SFV-infected individuals (S1 Table). Thirty-four (77%) neutralized the GI-D468 strain, with a median titer of 1:496 (range 1:10–1:14,724; Fig 1C). Twenty-two (50%) neutralized the GII-K74 strain, with a median titer of 1:20 (range 1:10–1:2,279, Fig 1C). Plasma samples from eight uninfected hunters living in the same villages as infected individuals (S1 Table) had titers < 1:20 against both strains (Fig 1C). The frequency of samples with neutralizing activity were significantly different between infected and uninfected hunters (Fisher’s exact test *P* values were < 0.0001 and 0.01 for the GI-D468 and GII-K74 strains, respectively).

The 44 individuals infected with gorilla SFV can be defined as single, dual, or non-neutralizers: 24 plasma samples neutralized a single strain, 16 neutralized both strains, and four had no neutralizing activity. The lowest dilution tested for all plasma samples was 1:20, due to limited sample volume for some participants. Low neutralization activity observed at a 1:20 dilution only was confirmed using lower plasma dilutions (1:10 and 1:5). Among the 40 reactive plasma samples, five had low to moderate titers (< 1:200), 24 had high titers (≥ 1:200 and < 1:2,000), and 11 had very high titers (≥ 1:2,000). Thus, most humans infected with a gorilla SFV produced high titers of antibodies that neutralized primary zoonotic strains circulating in the same geographical region.

### Plasma neutralization capacity matches the genotype of strains infecting single neutralizers

We hypothesized that the strain infecting each individual was the major determinant of genotype-specific neutralization. Among the 44 gorilla SFV-infected people, for whom neutralization assays were performed, 34 had had their *env* gene PCR-amplified and sequenced in a previous study [33]. Twenty-six SFV strains belonged to the GI genotype and eight to the GII genotype. Fig 1D and S2 Table show the neutralization titers against both strains and the genotype of the SFV strain for each participant: the infecting and neutralized strains were of the same genotype (15 GI, 5 GII) for all single neutralizers. Dual neutralizers were infected with strains from GI (n = 10), GII (n = 2), or undetermined (n = 4) genotype. Their neutralization titers against the GI-D468 and GII-K74 strains were not related: for example, plasma from individual BAK74 neutralized the GI-D468 strain more efficiently than its autologous strain (neutralization titers: 1:340 *vs* 1:62). The results at this point support a match between the specificity of neutralization and viral genotype for single, but not dual neutralizers.

### Half of dual neutralizers are coinfected with strains of both genotypes

We hypothesized that dual neutralizers were coinfected with strains from the two genotypes. We thus established two genotype-specific PCR assays to demonstrate coinfection. Eight people had blood cell DNA samples positive by both GI and GII-specific PCR, showing coinfection by strains of the two genotypes. Twenty-one people were infected with a GI strain only (19 previously classified as GI and two undetermined, S2 Table) and 10 with a GII strain only (seven previously classified as GII and three undetermined, S2 Table). The coinfection status was unknown for five individuals because we ran out DNA or both genotype-specific PCRs gave negative results. Re-examination of the neutralization pattern and results from genotype-specific PCR showed that one of the eight coinfected individuals neutralized a single strain, whereas seven neutralized both strains (Fig 1E, S2 Table). The nine other dual neutralizers tested positive by either GI- or GII-specific PCR only or were undetermined. In conclusion, coinfection by SFV strains of the two genotypes occurred in eight of 39 (20%) infected individuals, and most coinfected individuals raised antibodies that neutralized strains of both genotypes.

### Neutralizing epitopes are located in the variable region of the envelope protein

**Fig 2:**
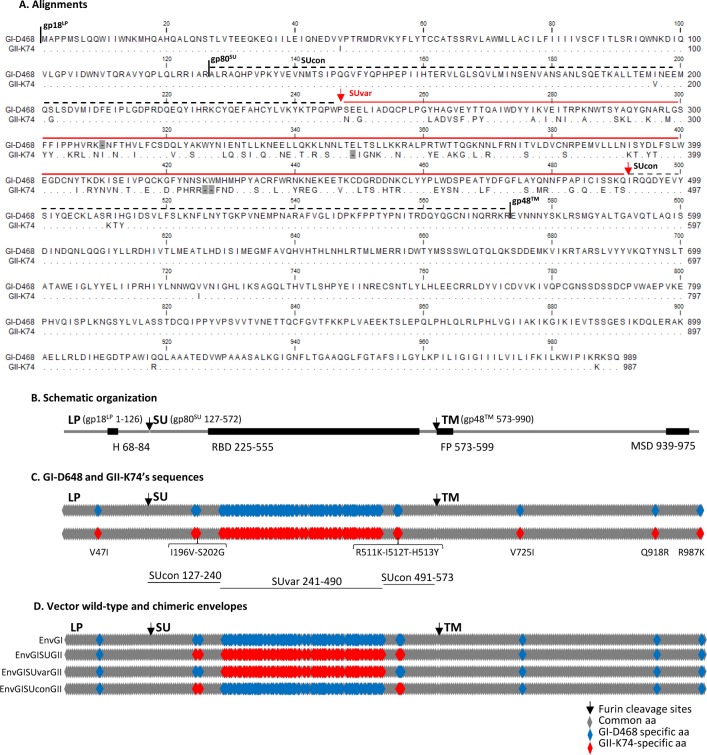
Gorilla SFV envelope proteins: alignment, schematic organization, localization of sequence variations, and design of the chimeric proteins used for epitope mapping. A. Env sequences from GI-D468 and GII-K74 strains were aligned using CLC Mainworkbench software. Identical residues are indicated with dots. The three subdomains (gp18^LP^, leader peptide; gp80^SU^, surface protein; gp48^TM^, transmembrane protein) are indicated above the sequences. The lines indicate the localization of the variable (SUvar, red) and conserved (SUcon, black) regions, respectively. B. Schematic organization of the SFV Env protein. Amino acid positions are those of the GI-D468 strain. Furin cleavage sites (arrows) and envelope subunits are indicated above the grey line. Major functional or structural domains are shown by the bold line (H, hydrophobic region; RBD, receptor binding domain; FP, fusion peptide; MSD, membrane spanning domain). C. Schematic representations of GI-D468 and GII-K74 Env sequences. Grey symbols represent identical amino acids and blue/red symbols GI/GII-specific amino acids, respectively. Sequences from point mutations outside SUvar are indicated below the diagram, as well as the SUcon and SUvar regions. The color code is not to scale in the SUvar region: amino acid identity is 58% in this region. D. Schematic representation of the Env proteins used to produce the vectors and map the neutralization epitopes. Full-length GI-D468 was used as the wild-type backbone, in which the SU, SUvar, and SUcon coding sequences from GII-K74 were inserted. The same color code is used as in panel B.

We next investigated the localization of the epitopes targeted by the antibodies. The match between genotype(s) from neutralized and infecting strains supports the direct interaction of genotype-specific amino acids (aa) with the neutralizing antibodies or their indirect involvement with the proper conformation of the epitopes. Alignment of Env protein sequences from the GI-D468 and GII-K74 strains showed that the central region of gp80^SU^ harbors a variable region (SUvar) which differs significantly in aa composition, with 42% (101 of 243) nonidentical aa (Fig 2A). In contrast, the surrounding bipartite conserved portion of the SU (SUcon) contains only 2.5% nonidentical aa: two N-terminal and a cluster of three C-terminal aa of the SUvar region. The gp18^LP^ and gp48^TM^ subunits vary by one and three aa, respectively, corresponding to less than 1% nonidentical aa.

We generated foamy virus vector particles (FVV) enveloped by wild-type GI Env (EnvGI) and chimeric GI/II Env with exchange of SU (EnvGI-SUGII), SUvar (EnvGI-SUvarGII), or SUcon (EnvGI-SUconGII) sequences using chimeric Env packaging constructs (Fig 2). Neutralization assays were performed with the FVV, using the same cell line (GFAB) and same moi (100 IU/well) as for the replicating viruses. Susceptibility to neutralization by human plasma samples of FVV with EnvGI and EnvGI-SUGII Env was similar to the one of replicating GI-D468 and GII-K74 viruses with less than a two-fold difference in neutralization titers (S1 Fig).

**Fig 3:**
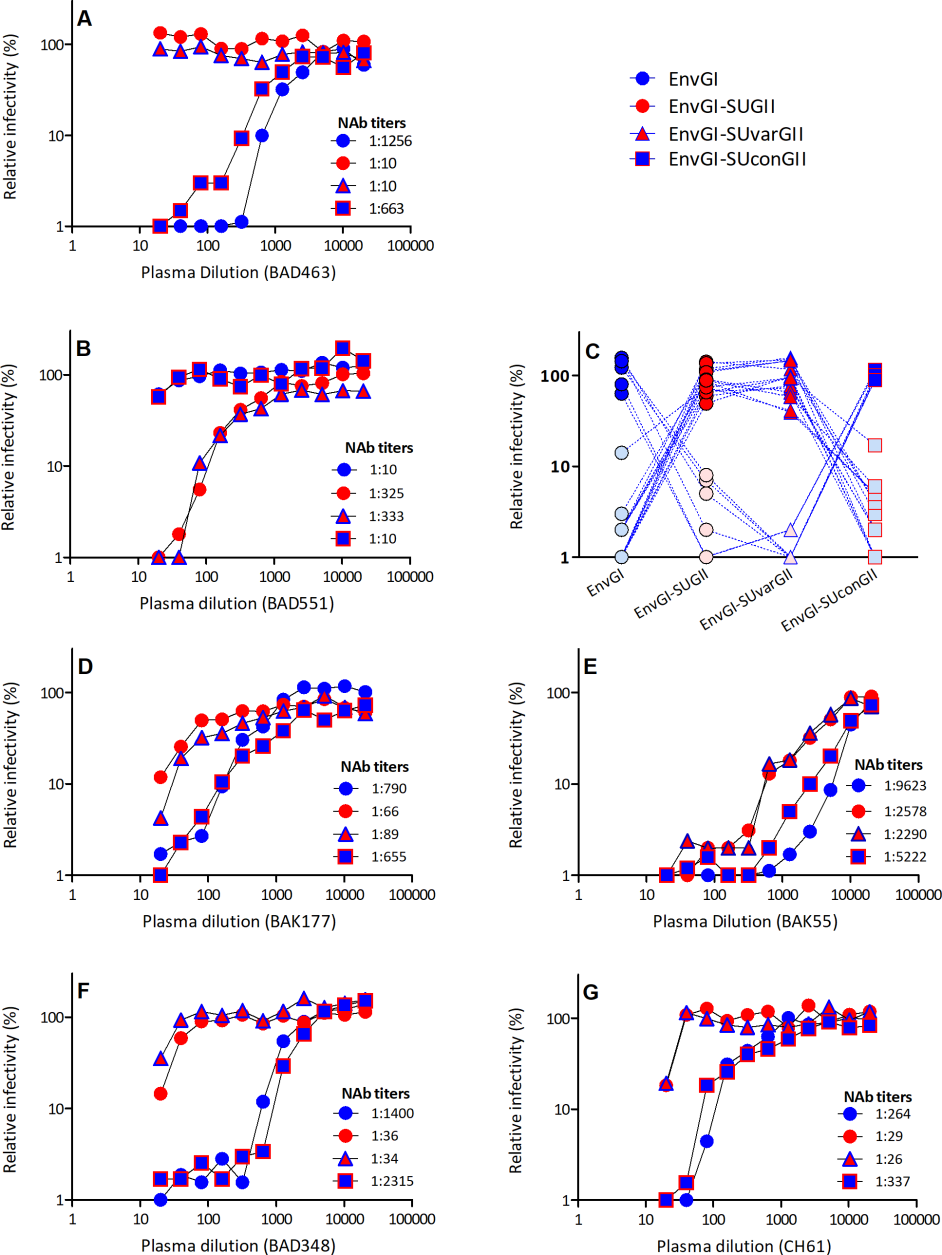
Neutralizing antibodies target the SUvar region. Neutralization assays were performed with plasma samples from gorilla SFV-infected individuals against the four FFV carrying wild-type EnvGI, chimeric EnvGI-SUGII, EnvGI-SUvarGII, and EnvGI-SUconGII. Cells were transduced with untreated FFV to provide the reference value. Relative infectivity was calculated for wells treated with plasma samples and is expressed as the percentage of the reference value. Panels A, B, and D through G present full titration curves for plasma from single neutralizers (A-B), coinfected dual neutralizers (D-E), and dual neutralizers without molecular evidence of coinfection (F-G). Panel C shows the relative infectivity after incubation with plasma samples from single neutralizers diluted 1:80. Fourteen plasma samples were specific for GI-D468 and are presented with dark blue/light pink symbols; six plasma samples were specific for GII-K74 and are presented with light blue/red symbols.

We first tested samples from single neutralizers: plasma sample from individual BAD463, which neutralized the GI-D468 virus only, efficiently neutralized the vectors carrying EnvGI and chimeric EnvGI-SUconGII, but not chimeric EnvGI-SUGII or EnvGI-SUvarGII (Fig 3A). Plasma BAD551, which neutralized only the GII-K74 virus, gave the opposite pattern of neutralization (Fig 3B). Thus, neutralization epitopes recognized by these two plasma samples are encoded by the SUvar region. These results were confirmed, with 14 plasma samples neutralizing the GI-D468 virus only and six neutralizing the GII-K74 only: when diluted at 1:80, the plasma samples reduced the relative infectivity of vectors expressing the corresponding SUvar region by five to 100-fold (Fig 3C).

We next tested plasma from individuals coinfected with strains of two genotypes against the four vectors. Neutralization titers depended on the expression of the SUvar region. For example, plasma from individual BAK177 had a high neutralization titer against the GI-D468 virus (1:1,025) and a low titer against the GII-K74 virus (1:36). Its neutralization titers against vectors carrying EnvGI and EnvGI-SUconGII were high (1:790 and 1:655) and those against EnvGI-SUGII and EnvGI-SUvarGII vectors were low (1:66 and 1:89) (Fig 3D). Other plasma samples showed similar neutralization patterns (Fig 3E and S3 Table). In conclusion, the SUvar domain carries the neutralizing epitopes recognized by antibodies from single neutralizers and coinfected individuals.

The plasma of nine individuals neutralized both viral strains, whereas they were only positive in a single genotype-specific PCR. There are two possible explanations for this result: they were coinfected, but only a single strain was detectable by PCR, or their neutralizing antibodies target epitopes that are located in regions conserved between the two genotypes. For most, the titers were clearly different against the two replicative viruses (Fig 1E), arguing against the neutralization of conserved epitopes. Indeed, titers depended upon expression of the SUvar region by vector particles, as shown in Fig 3F and 3G and S3 Table. Thus, dual neutralizers were most likely coinfected by strains of two distinct genotypes.

### Neutralization activity is detected in the plasma of all SFV GI-infected and most SFV GII-infected individuals

Four of 18 individuals infected with a GII strain did not neutralize the GII-K74 strain (three were infected with a GII strain only and one was infected with GI and GII strains but neutralized the GI-D468 strain only). One individual had no neutralizing activity against either strain and was confirmed to have been infected on the basis of a Gag-specific WB and an LTR-specific PCR only, whereas the Pol, Env, and Env genotype-specific PCR assays (this paper) were negative [8, 33]. Of note, all 31 individuals infected with a GI strain were able to neutralize the GI-D468 strain. In conclusion, neutralizing antibodies were detected in the plasma of 100% GI SFV-infected individuals and 78% of those infected by GII SFV.

We therefore investigated whether the higher proportion of non-neutralizers among GII-infected individuals was related to differences in Env sequence variability across strains. Env sequences derived from genotype-specific PCRs amplicons were aligned for the 31 GI and 18 GII strains infecting our study population and the viral strains used in the neutralization assays, with sequences ordered as a function of their neutralization titers (S2 Fig). Sequence variability was similar for the GI and GII strains: 16% and 18% of the positions had at least one aa change among the analyzed GI and GII sequences, respectively. Analysis of 12 full-length GI and seven full-length GII Env sequences showed similar variability [33]. Thus, there was no clear relationship between Env protein diversity within each genotype and the detection of neutralizing antibodies.

Among the four GII-infected non-neutralizers, individual CH101 had full length *env* gene sequence data available. There was a single nonconservative F_420_Y change in the CH101 SUvar sequence, using the GII-K74 sequence as a reference. In an ELISA assay, plasma from CH101 did not react with peptides corresponding to GII-K74 and CH101 amino acids 411 to 430, whereas plasma from BAK74 recognized both peptides. Thus, the antigenic difference between the infecting and neutralized strains does not explain the lack of neutralization, at least for individual CH101. We were unable to obtain a second sample from all non-neutralizers to confirm the absence of plasma neutralization and amplify the *env* gene.

### Neutralizing antibodies frequently cross-recognize gorilla and chimpanzee SFV with phylogenetically related SUvar domains

The formerly used classification of viral strains was based on their susceptibility to neutralization (serotyping) and showed segregation of SFV according to the host species. Among SFV infecting African NHPs, those from *chlorocebus*, *papio* and chimpanzee belong to four distinct serotypes, with chimpanzee SFV strains divided into two [21]. Gorilla SFV had not been previously typed. We therefore examined the susceptibility of SFV strains to neutralization according to their origin. We started by testing two laboratory-adapted chimpanzee SFV strains with defined serotypes: PFV, belonging to the CI genotype and serotype 6, and SFV7, belonging to the CII genotype and serotype 7 [34]. Plasma samples from eight individuals infected with a chimpanzee SFV were tested and neutralized CI-PFV (n = 3), CII-SFV7 (n = 6), GI-D468 (n = 6) or GII-K74 (n = 4) (Table 1). The plasma from seven individuals neutralized a single chimpanzee SFV and one was a dual neutralizer.

**Table 1:** Neutralization specificity of plasma samples from chimpanzee SFV-infected hunters.

Code	Genotype	1:titer			
		GI-D468	GII-K74	CI-PFV	CII-SFV7
AG15	CII	25	171	10	837
BAD316	CII	209	670	10	1622
BAD327	CII	31	27	10	277
CH66	CII	715	10	343	10
H3GAB56	CII	10	10	10	55
H4GAB59	CII	10	10	10	185
PYL106	CI	1451	10	2994	10
PYL149	Not determined	1040	52	565	103

**Fig 4:**
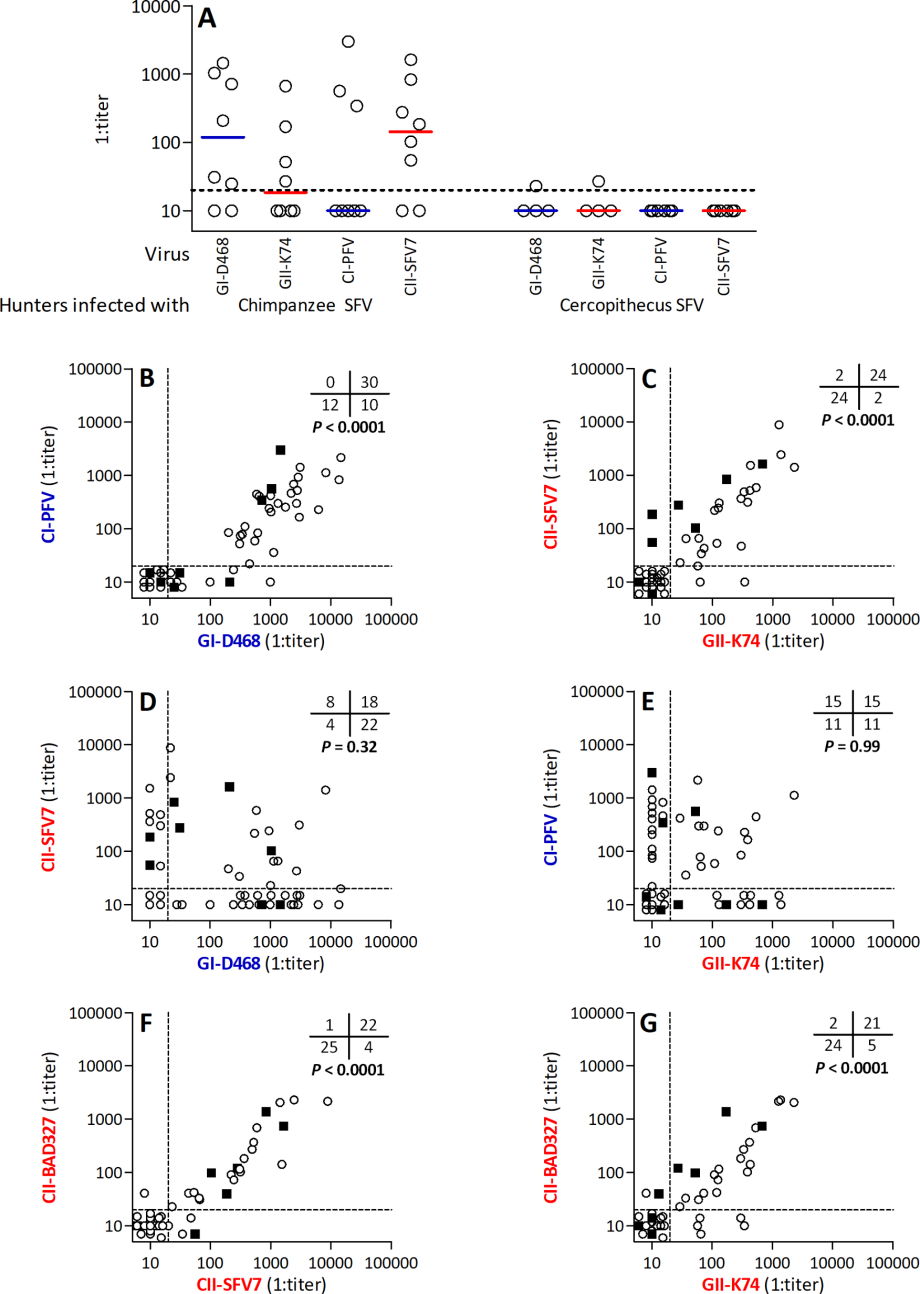
Frequent cross-neutralization of gorilla and chimpanzee SFV belonging to homologous genotypes. Neutralization assays were performed against four viral strains with plasma samples from individuals infected with three SFV species (chimpanzee, gorilla, and cercopithecus). A. Neutralization titers against four strains (GI-D468, GII-K74, CI-PFV, CII-SFV7) are shown for individuals infected with chimpanzee or *cercopithecus* SFV; bars indicate the median values. B to G: Neutralization titers against pairs of strains are presented with symbols corresponding to the infecting SFV species (open circles: gorilla SFV; filled squares: chimpanzee SFV). The dashed lines indicate neutralization detection thresholds (1:20). Tables indicate the number of individuals with and without neutralizing antibodies against each of the two strains, with *P* values from Fisher’s exact test.

We further characterized the cross-neutralization of gorilla and chimpanzee SFV by testing the plasma of the 44 gorilla SFV-infected individuals: 17 neutralized CI-PFV only, eight CII-SFV7 only, 10 both strains, and nine none of them (S2 Table). In contrast, plasma from *cercopithecus* SFV-infected individuals had no or low neutralization titers against gorilla and chimpanzee SFVs (Fig 4A). Thus, gorilla and chimpanzee SFV share at least some neutralizing epitopes, but are not inhibited by plasma from hunters infected with monkey SFV.

Combining the data from gorilla and chimpanzee SFV-infected individuals (n = 52 samples), neutralization of homologous strains (*i*.*e*. phylogenetically related SUvar domains) were mostly concordant. Neutralization of GI-D468 and CI-PFV were concordant for 42 samples (12 negative and 30 positive) and discordant for 10 samples, which neutralized only GI-D468 (Fig 4B). In reactive plasma samples, titers against GI-D468 and CI-PFV were correlated (Spearman’s ρ = 0.696, P < 0.0001). The second genotype showed a similar profile (Fig 4C): neutralization of GII-K74 and CII-SFV7 were concordant for 48 samples (24 negative and 24 positive), and discordant for four, which neutralized either GII-K74 (n = 2) or CII-SFV7 (n = 2) viruses. In reactive plasma samples, titers against GII-K74 and CII-SFV7 were correlated (ρ = 0.823, *P* < 0.0001). Neutralization titers against viruses with unrelated SUvar domains were not correlated (*P* = 0.32 for GI-CII and *P* = 0.99 for GII-CI, Fig 4D and 4E). These data confirm that the SUvar domain is a major determinant of recognition by neutralizing antibodies raised against both gorilla and chimpanzee SFVs.

We compared the neutralization of the laboratory-adapted CII-SFV7 strain to that of zoonotic primary chimpanzee SFVs that we isolated from Cameroonian hunters [12]. Both CII-D327 and CII-AG15 belonged to the CII genotype and neutralization titers against both viruses were strongly correlated (Spearman’s rho = 0.932, *P* = 0.0002). CII-D327 was neutralized by plasma from 23 of 52 individuals infected with either gorilla or chimpanzee SFV. Neutralization of the CII-D327 and both CII-SFV7 and GII-K74 strains was mostly concordant, and in reactive plasma samples, the titers were strongly correlated (Fig 4F and 4G). Conversely, neutralization of CII-D327 was unrelated to that of CI-PFV and GI-D468 (Fisher’s exact test *P* = 0.26 and *P* = 0.33, respectively). In conclusion, laboratory-adapted and primary SFV strains show similar susceptibility to neutralization. Nevertheless, the occurrence of discordant responses against strains belonging to the same genotype support some inter-individual variability in the interactions between neutralizing antibodies and susceptibility sites on SFV Env.

### Higher breadth of neutralizing antibodies is associated with smaller hematological changes in SFV-infected individuals

**Fig 5:**
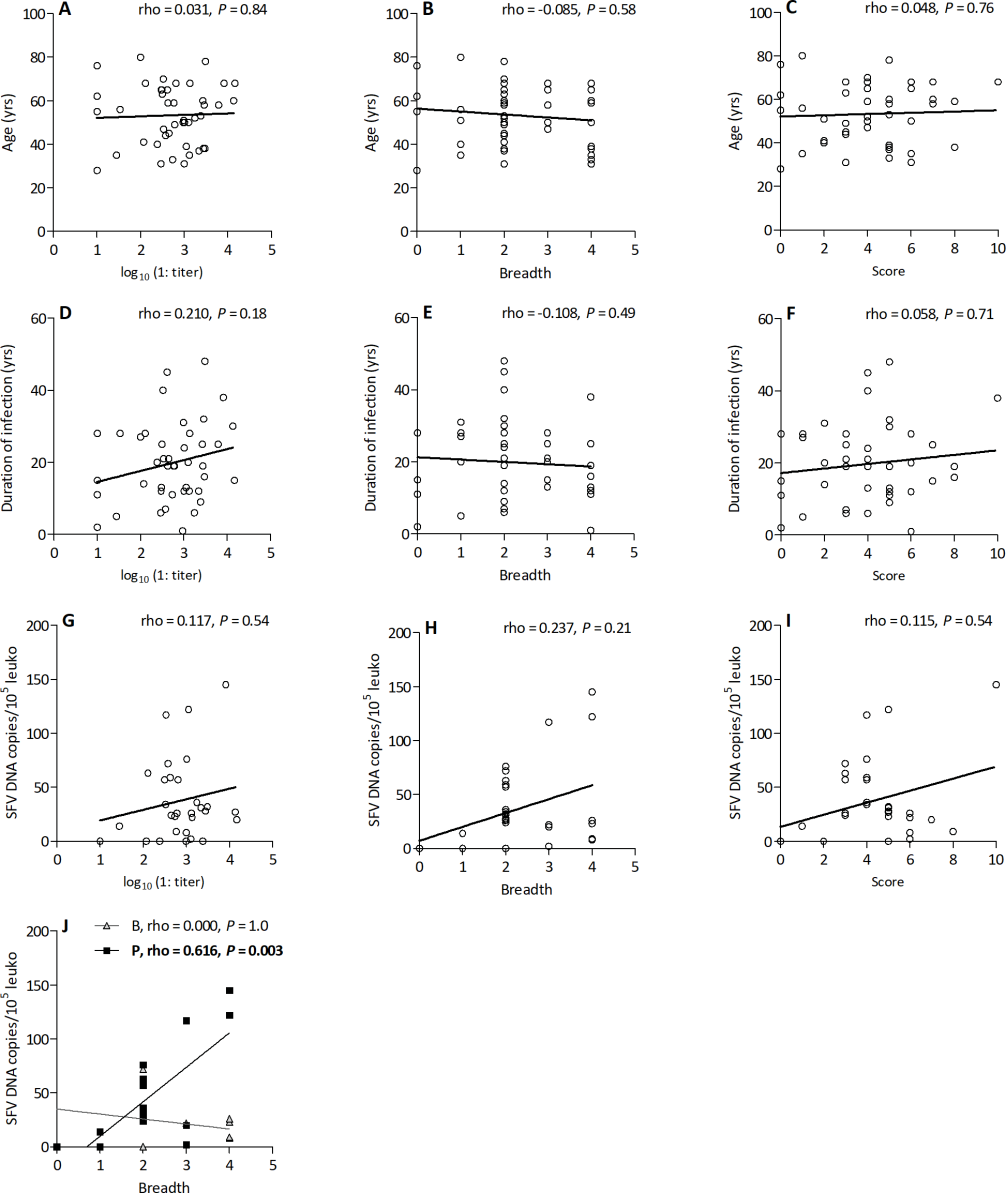
Magnitude and breadth of neutralizing antibodies and parameters of SFV infection Correlations between quantitative measures of neutralization and parameters of infection are presented. The neutralization titer against the homologous SFV strain was used; for coinfected individuals we considered the highest of the two titers. Breadth was defined as the number of neutralized strains of the four tested. For each viral strain, a score was assigned based on the neutralization titer (neutralization titers < 20 and those ranging from 1:20 to 1:200, 1:200–1:2000, and > 1:2000 correspond to 0, 1, 2 and 3 points, respectively). The sum of these points for the four tested strains defined the neutralization score of a plasma sample. Age (A, B, C), duration of infection (D, E, F), and SFV DNA levels (G, H, I) are presented as a function of the neutralization titers (log_10_ transformed, A, C, and E), breadth (B, D, and F), and neutralization score (C, F, I). The lines represent the linear regression curves. J: SFV DNA levels are presented as a function of the neutralization breadth for Bantus (triangles) and Pygmies (squares). Results from the Spearman rank test are indicated on the graphs and statistically significant results are shown in bold.

We investigated whether neutralizing antibody levels were related to the parameters of SFV infection. The neutralizing activities of plasmas was tested against four SFV strains, corresponding to the two genotypes from each of the two host-specific SFV clades (GI-D468, GII-BAK74, CI-PFV and CII-SFV7). We considered two quantitative measures of neutralization: magnitude, defined as neutralization titers against the species- and genotype-matched strains and breadth, defined as the number of neutralized strains. Neither magnitude nor breadth of neutralizing antibodies were associated with age, duration of infection, or blood SFV DNA levels (Fig 5). Neutralization titers varied over a wide range, leading us to consider a composite measure to better quantify the neutralization capacity of plasma samples against the panel tested. A score was assigned for each plasma sample and each viral strain, based on neutralization titers. Neutralization titers < 20, ranging from 1:20 to 1:200, 1:200–1:2000, and > 1:2000 correspond to 0, 1, 2, and 3 points, respectively [35]. The sum of the points for the four tested strains defined the neutralization score of a plasma sample. The neutralization score was not associated with age, duration of infection, or blood SFV DNA levels (Fig 5C, 5F and 5I). The duration of infection was different for the two ethnic groups, Bantus and Pygmies (medians: 13 and 21 yrs for Bantus and Pygmies, respectively, Mann-Whitney test *P* = 0.02), and SFV DNA levels tended to differ (22 and 32 copies/10^5^ cells, *P* = 0.09). Bantus and Pygmies had similar neutralization magnitude (Medians: 1:395 vs. 1:965, *P* = 0.11) and breadth (Medians: 2 vs; 2, *P* = 0.68). In Pygmies, higher breadth was associated with higher SFV DNA levels (Fig 5J), whereas the SFV DNA loads were very homogeneous and not related to the neutralization breadth in Bantus (Fig 5J). Neutralization titers against the four viruses were stable for samples collected from seven individuals at two time points, one to seven years apart (Table 2).

**Table 2:** Stability of NAb titers in the plasma of infected individuals.

Code	Year of sampling	1:titer			
		GI-D468	GII-K74	CI-PFV	CII-SFV7
BAD348	2013	2460	10	238	10
	2015	1006	29	258	10
BAD447	2013	587	529	68	588
	2015	345	576	62	441
BAD463	2011	536	10	33	10
	2013	486	10	55	10
BAD468	2010	2068	47	237	66
	2013	2865	—^a^	223	47
BAK177	2013	1125	77	88	— ^a^
	2015	1057	177	215	371
BAK242	2010	418	10	82	10
	2011	610	10	83	10
BAK40	2008	25	10	10	10
	2015	70	10	20	10

^a^ not tested

**Fig 6:**
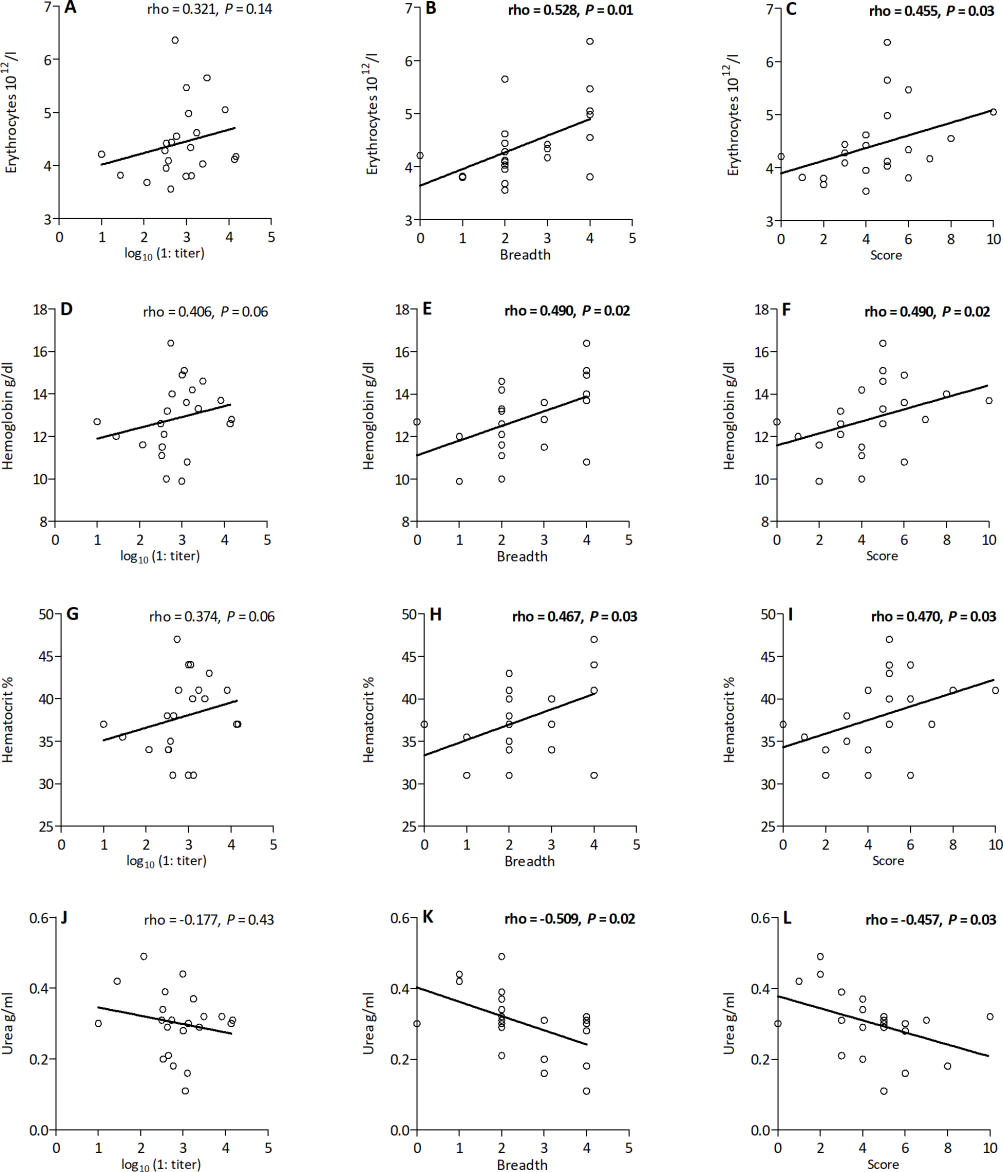
Neutralization magnitude, breadth and score are associated with hematological variables Correlations between quantitative measures of neutralization and hematological parameters are presented. Erythrocyte counts (A, B, C), hemoglobin (D, E, F), hematocrit (G, H, I), and blood urea (J, K, L) levels are presented as a function of neutralization titers (A, D, G, and J), breadth (B, E, H, and K), and potency (C, F, I and L). The lines represent the linear regression curves. Results from the Spearman tests are indicated on the graphs and statistically significant results are shown in bold.

Finally, we searched for associations between neutralization levels and hematological parameters, of which the levels were significantly different between SFV-infected individuals and matched uninfected controls [32]. Hunters infected with SFV had lower hemoglobin levels than uninfected hunters. Here, among 22 SFV-infected individuals, those with a higher neutralization breadth or score had higher hemoglobin levels, as well as a higher hematocrit and erythrocyte levels (Fig 6A–6I). Higher neutralization magnitude also tended to be associated with higher hemoglobin levels and hematocrit. Only 33% of individuals that neutralized three or four strains had mild or moderate anemia (hemoglobin level < 13 g/dl), whereas as the frequency was 69% for those who neutralized two strains or less. In addition, SFV-infected individuals had higher urea levels than uninfected controls, which were lower for those with a higher neutralization breadth or score (Fig 6K and 6L). We also repeated the analyses with a higher neutralization cut-off (1:80) and observed similar associations or trends for associations. Analyses stratified by ethnic group showed similar associations in both groups, except for a strong correlation between neutralization breadth and protein levels in Bantus only (Spearman’s rank test rho = 0.874, *P* = 0.01). In conclusion, individuals who produced neutralization antibodies with a larger breadth displayed higher hemoglobin and lower urea levels than those with a narrower neutralization range.

## Discussion

We report the presence of high titers of neutralizing antibodies in the plasma of most SFV-infected individuals. Ape SFV species comprise two genotypes that cocirculate in humans and NHPs from Central-Africa [33]. We detected predominantly genotype-specific neutralization. We constructed vectors with chimeric Env, based on naturally occurring sequence variations, and used them to map dominant viral susceptibility sites to the dimorphic portion of the surface domain that overlaps the receptor-binding domain. The frequent cross-neutralization of gorilla and chimpanzee SFVs supports the recognition of highly conserved epitopes. Our description of the neutralization patterns of SFV-infected humans provides two key pieces of information concerning their virological status: gorilla SFVs appear to continuously or sporadically express their viral proteins and the coinfection rate is over 30%. Several hematological markers differ between SFV-infected individuals and uninfected controls [32]. We found that a larger neutralization breadth is statistically associated with smaller hematological changes, supporting the beneficial impact of the humoral response on the clinical outcome of infected hosts. SFV has a high capacity to cross the host-species barrier and persistently infect humans. Overall, our data demonstrate potent neutralization, targeting mostly conserved and immunodominant epitopes located in the receptor binding domain, produced by approximatively 90% of gorilla SFV-infected humans. These properties may have helped to block the spread of SFV in the human population.

We show here that most of the 52 individuals infected with gorilla or chimpanzee SFV produced neutralizing antibodies. Titers varied by over 1,000-fold between participants and were high or very high in 75%. A key element of the cross-species transmission of viruses is their replication in the new host. A side-by-side comparison of SFV viral load, replication sites, and immune response in human and animal samples is impossible to perform for apes living in the wild. However, neutralization titers (< 1:20 to 1:15,000) in human plasma were similar to those reported in chimpanzee and gorilla samples (< 1:32 to 1:2,500) tested against chimpanzee SFV [36]. SFV-specific IgG was found to be slightly lower in occupationally infected humans than in captive chimpanzee samples using a different assay of SFV-specific antibodies (semi-quantitative WB assay) [37]. A humoral response of comparable magnitude in humans and apes reflects the lack of an intrinsic restriction of SFV replication *in vivo* in humans, in accordance with the lack of *in vivo* virus adaptation after a zoonotic infection [12].

The humoral response in gorilla SFV-infected people is consistent with persistent exposure to viral antigens during chronic viral infection, as neutralization titers were (1) high in most individuals; (2) of similar magnitude among people who have been chronically infected for 1 to > 40 years; and (3) stable over a one to seven year-long longitudinal follow-up. Some Brazilian workers with serological evidence of infection by New World Monkey (NWM) SFV were shown to lose their seroreactivity in western-blot assays [38]. Blood SFV DNA levels for NWM strains are usually undetectable in humans, in contrast to NHP [38–40]. Overall, both the frequency of SFV DNA detection in human blood samples and antibody levels support higher expression for SFV that originate from the closest human relatives [8, 38, 39, 41]. In conclusion, high neutralization titers in humans infected with ape SFV strongly suggest that these zoonotic viruses express their proteins in their new human hosts.

We describe the paradoxical situation of high neutralization titers, an indicator of viral protein expression, and undetectable viral RNA in blood and saliva of the same study population [14]. Blood antibodies reflect viral protein expression in the organism from months to years, whereas qRT-PCR assays are limited to the tissue analyzed (blood or buccal samples), at a given time point. Thus, the lack of SFV RNA detection in blood or saliva does not prove the absence of viral replication in the human hosts. Furthermore, circulating lymphocytes—the major blood cell type carrying SFV DNA [13]–are usually in a resting state not permissive to SFV replication, whereas proliferating lymphocytes are mostly located in lymphoid organs and tissues. *In vitro*, SFV latency is characterized by persistent expression of the Bet protein only, in the absence of Env expression [42–44]. Thus, the presence of high levels of Env-specific antibodies provide indirect evidence against *in vivo* latency of gorilla SFV in humans.

We recently observed that several hematological parameters differ between SFV-infected hunters and age-matched uninfected hunters living in the same area [32]. The hematological alterations consisted of reduced hemoglobin and red-cell levels and elevated protein, urea, creatinine, creatine phosphokinase, and lactate dehydrogenase levels. Individuals with a large neutralization breadth had smaller hematological alterations than those with a narrow neutralization breadth. Neutralization breadth depends upon the targeted epitopes, diversification of viral sequences in the infected host, efficient cross-talk between B and T lymphocytes, and the maturation and selective expansion of high affinity antibodies. If the neutralization breadth of SFV-specific antibodies reflects their *in vivo* efficacy, as shown for other viral infections [45], our results may reflect the protective action of neutralizing antibodies against hematological changes observed in SFV-infected individuals.

Approximately 10% of gorilla SFV-infected individuals had detectable neutralizing antibodies. We were unable to obtain a second blood sample from these individuals, precluding confirmation of the diagnosis and the neutralization pattern. For one non-neutralizer, CH101, the sequence variation between the autologous virus and the strain used in the neutralization assay is unlikely to explain the negative results. Another non-neutralizer, BAK235, was included in our recent clinical study [32]. He was not immunosuppressed, and his hematological parameters were close to the median value of the group (Fig 6). All participants were apparently healthy at the time of sampling and all had negative HIV serological assays. Thus, undetectable neutralization was probably not a consequence of poor health. The lack of neutralizing antibodies may be related to low viral load, as suggested by undetectable SFV DNA in the qPCR assay for individual BAK235 [8], and failure to amplify the *env* gene for individuals BAK235, CH86, and H10GAB79. We indeed observed a positive correlation between antibody titers and blood SFV DNA levels for Pygmies. Overall, undetectable plasma neutralization in some participants may be explained by low viral replication.

Here, we showed that neutralizing epitopes targeted by human plasma are mostly located in the SU domain of the Env protein and demonstrated the relation between the genetic and antigenic properties of gorilla SFV strains. For chimpanzee SFV, the initial description of two serotypes [46] is consistent with the latter demonstration of infection of these NHP species by these two genotypes [33]. Here, we directly demonstrated that chimpanzee SFV strains belonging to serotypes 6 and 7 [36, 46] were neutralized by the plasma of individuals infected with CI and CII genotypes, respectively. Feline foamy virus (FFV) genotypes were shown to determine the type-specificity of neutralizing antibodies [47] and the fragment of the genome comprising the *env* and *bel1* genes to carry the determinants for FFV genotype-specific neutralization [48, 49]. The two genotypes of FFV are present in domestic and wild felids of various geographic regions [50], and several Asian and African NHP species are infected by SFV strains segregating into two genotypes [33]. Overall, targeting of the bimorphic region located in the Env protein surface domain is a cardinal feature of antibodies that neutralize thus far studied foamy viruses.

Most plasma samples neutralized both gorilla and chimpanzee SFV, indicating high conservation of some of the sites targeted by the neutralizing antibodies. The chimpanzee/gorilla split occurred eight million years ago [51]. The phylogeny of the conserved and variant portions of the *env* gene differ: for the central SU region, SFVs form two clades, corresponding to the two genotypes, and within each clade, isolates are distributed according to their host [33]. Consequently, aa identity of the SUvar region is ≈ 70% for GI-CI and GII-CII pairs of viral strains, and only ≈58% for GI-GII and CI-CII pairs. In the conserved backbone of Env, aa identity is ≈ 78% for GI-CI and GII-CII pairs and above 95% for GI-GII and CI-CII pairs. These data are fully concordant with the cross-neutralization of gorilla and chimpanzee SFVs for strains from the same genotype group. However, some plasma samples only neutralized strains from a single host species (19% and 8% between GI-CI and GII-CII strains, respectively). Such a narrow pattern may reflect inter-individual variation in Env sequences and/or antibody binding sites. Minor antigenic differences between serologically related strains have indeed been detected for FFV and chimpanzee SFV [34, 52]. The genetic stability of foamy viruses in each host and among hosts of the same species limit their potential to escape neutralizing antibodies. In humans, the genetic stability of SFV is high (98.6 homology in the Pol protein in blood samples taken four years apart [15]), and no adaptation to the human host has been described, even decades after infection [12, 53]. Viruses from human-NHP transmission pairs had almost identical sequences [15, 54]. Overall, neutralization epitopes are expected to be conserved because of the previously reported genetic stability of SFV and we further demonstrate their conservation during the coevolution of SFV with their NHP hosts.

Physical blockade of the interaction between viruses and their cellular receptor is an important and common mechanism of virus neutralization [55]. The SFV receptor is unknown, but ubiquitous. Interference experiments showed that simian, feline, bovine, and equine FV share the same receptor [56]. The particle-associated envelope glycoprotein of FV is composed of trimers arranged in interlocked hexagonal assemblies, with the SU bearing the receptor binding site located at the top of the spikes [57]. Here, we demonstrated that immunodominant neutralizing epitopes are predominantly located in the dimorphic central part of the SU that overlaps the receptor binding domain [58]. Polymorphic aa located outside of the SUvar region are not involved in recognition by neutralizing antibodies, nor required for the proper conformation of Env, as chimeric Env built for this project mediated the entry of vector particles. The suppression of viral transmission by effective neutralizing antibodies may induce evolutionary pressure that selects antibody escape mutations but immune evasion may be limited if it results in variant viruses with impaired replicative capacity. The Env sequence from SFV is less variable than that of Gag [59]. This pattern is opposite to that observed for most other retroviruses and probably reflects strong structural constraints on the Env protein, which in turn may favor the efficient neutralization of SFVs.

One third of gorilla SFV-infected individuals were dual neutralizers and one fifth had evidence of coinfection by strains from the two genotypes. All but one coinfected individual were dual neutralizers. Discordance between serological and molecular assays for genotype-specific SFV detection may have several causes. Nucleic acid tests give negative results when the viral load is low or strains carry variant nucleotides in the primer sequences. The sensitivity of serological detection of an infection rarely reaches 100%, due to the lack of an antibody response in some individuals and/or minor antigenic differences between viruses. Here, we only detected coinfection by strains belonging to distinct genotypes, and true coinfection rates may be higher than those estimated by serological or molecular detection of viral genotypes.

The rate of coinfection in Cameroonian and Gabonese hunters is in the same range as that reported in Bangladeshi infected with macaque SFV [60]. Dual neutralization has been reported in sera drawn from chimpanzees housed in a primate research center, as well as bush chimpanzees [46, 61] and FFV-infected cats [47, 62]. In Bangladesh, the analysis of viral sequences from macaques revealed that at least one of four adult animals was infected with at least two strains [60]. Single genome amplification of wild chimpanzee samples has provided evidence of coinfection with genetically diverse viruses [63, 64]. In a wild community of chimpanzees in Ivory-Coast superinfection rates increased with age and were above 80% in adult animals [65]. Being injured while hunting apes in Africa is infrequent, well-recalled, and occurred more than once for only one individual of our study group. The high rate of coinfection in gorilla SFV-infected hunters is most probably the result of simultaneous transmission of several strains during a single exposure event. Indeed, we tested a series of ape samples and found that 26% of chimpanzees and 37% of gorillas from Cameroon and Gabon were coinfected by strains of both genotypes (S4 Table).

SFV coinfection is thus frequent in animals, and transmission of more than one virus strain to humans is frequent for macaque and gorilla SFV. Recombination is efficient *in vitro* [66] and has been detected in viruses infecting chimpanzees and macaques [60, 63]. Our results concerning coinfection are particularly relevant for the emergence of retroviruses in the human population, as simian immunodeficiency viruses have evolved through several recombination events in NHP hosts coinfected by two strains, progressively acquiring viral proteins able to antagonize host restriction factors, eventually allowing successful emergence of HIV-1 [9, 67, 68].

In conclusion, most SFV-infected humans produce neutralizing antibodies at levels similar to those detected in simian hosts. SFV-specific neutralizing antibodies display key features of protective responses: they target conserved epitopes and their breadth is associated with reduced biological changes in the infected hosts. The receptor-binding domain is the immunodominant region targeted by neutralizing antibodies. Whether subdominant cross-reactive antibodies recognizing the SUvar domain and/or antibodies targeting the conserved SU regions are present in plasma samples is still an open question. The overall robust SFV-specific neutralization calls for further studies dedicated to fine epitope mapping, Fc-mediated antiviral functions, and molecular characterization of human monoclonal antibodies [69–72].

## Materials and methods

### Ethics statement

Participants gave written informed consent. Ethics approval was obtained from the relevant national authorities in Cameroon (the Ministry of Health and the National Ethics Committee) and France (the Commission Nationale de l'Informatique et des Libertés (CNIL) and the Comité de Protection des Personnes Ile de France IV).

### Study participants

Field studies were performed on adult populations living in villages and settlements across rural areas of the rainforest in Cameroon and Gabon [8, 10, 31]. SFV infection was diagnosed by a clearly positive Gag doublet on Western blots, using sera from the participants, and the amplification of the *integrase* gene and/or LTR DNA fragments by PCR, using cellular DNA isolated from blood buffy-coats [8]. The origin of the SFV was identified by phylogenetic analysis of the sequence of the *integrase* gene, as described [8]. Plasma samples from 66 participants were used for this study, 53 Cameroonians and 13 Gabonese (S1 Table). Eight participants were not infected with SFV, eight were infected with a chimpanzee SFV, 44 with a gorilla SFV, and six with a *cercopithecus* SFV. All but four SFV-infected participants had been injured by a NHP. All participants were male, 37 were Bantus and, 29 were Pygmies. Their median (interquartile range, IQR) age was 53 (43–61) years. The duration of infection was estimated as the time elapsed between receiving the wound and the sampling and was 19 (11–28) years. Blood SFV DNA levels were determined by qPCR carried out on buffy-coats from 30 of the 44 gorilla SFV-infected participants, as described in a previous study [8]. Blood tests were carried out at the Centre Pasteur du Cameroun in Yaoundé for 22 individuals [32].

### Cells and viruses

Gorilla FAB (GFAB) cells are baby hamster kidney (BHK)-21-derived clones containing the β-galactosidase gene under the control of the LTR from SFVggo-hu.BAK74 LTR [73]. They were cultured in DMEM-5% fetal bovine serum (FBS) supplemented with 300 μg/ml G418 (Sigma-Aldrich, Lyon, France). Human embryonic kidney 293T cells (Cat. N° 12022001, Sigma) were cultured in DMEM-10% FBS.

Virus stocks were produced by infection of subconfluent BHK-21 cells that were passaged twice a week. Cultures displaying an extensive cytopathic effect were lysed by three freeze-thaw cycles. Cell lysates were clarified, filtered (0.45 μm pore size), and stored as single-use aliquots. Titrations were performed as described [73]. Briefly, serially diluted viral solutions were incubated with indicator cell lines in 96-well plates. Cells were fixed after 72 h with 0.5% glutaraldehyde in phosphate-buffered saline (PBS) for 10 min at room temperature (RT). Cells were washed with PBS and incubated for 1 h at 37°C with an X-Gal staining solution [2 mM MgCl_2_, 10 mM potassium ferricyanide, 10 mM potassium ferrocyanide, and 0.5 mg/ml 5-bromo-4-chloro-3-indolyl-B-D-galactopyranoside in PBS]. An S6 Ultimate UV Image analyzer (CTL Europe, Bonn, Germany) was used to count X-Gal stained cells. One infectious unit (IU) was defined as a blue cell or syncytia.

Original and first passage cell lysates were used to produce the viral stocks from zoonotic primary strains isolated from infected hunters [12]. The genotypes were defined in [33] and are abbreviated as follows: GI and GII for gorilla SFV genotypes I and II, respectively; CI and CII for chimpanzee SFV genotypes I and II, respectively. SFVggo_huBAD468 (JQ867465) and SFVggo_huBAK74 (JQ867464) belonged to the GI and GII genotype, respectively; SFVptr_huAG15 (JQ867462) and SFVptr_huBAD327 (JQ867463) belonged to the CII genotype. Two laboratory-adapted chimpanzee SFV isolates, SFVpsc_huPFV (KX087159, [74]) and SFVpve_Pan2 (SFV-7, *env* gene KT211269) belonged to the CI and CII genotypes, respectively. Here, strain nomenclature [75] was replaced by short names summarizing the genotype and strain names. For example, in the short name “GI-D468”, GI stands for GI genotype and -D468 for SFVggo_huBAD468.

### Plasma samples and neutralization assays

Human blood samples were collected into EDTA tubes and processed within 24 h of collection using standard techniques. Human plasma samples were stored at -80°C. Before use in neutralization assays, plasma samples were diluted 1 to 10 in DMEM + 1 mM MgCl_2_, heated 30 min at 56°C, to inactivate complement proteins, and frozen as single-use aliquots. Serial two-fold dilutions of plasma samples were incubated with SFV isolates for 2 h at 37°C before quantification of residual viral infectivity using the SFV microtitration assay in 96-well plates described above. The same multiplicity of infection (100 IU/well) was used for all viral strains. Assays were performed in triplicate. Neutralization titers were defined as the inverse of the plasma dilution required to reduce viral infectivity by half.

The lowest plasma dilution routinely tested was 1:20 to spare samples and avoid nonspecific reactivity. Titration curves were considered valid if there was a steady rise in the number of infectious units per well with increasing dilution of the sample and if a plateau was observed at the highest plasma dilutions. Neutralization titers were defined as the inverse of the dilution required to reduce viral infectivity by half. For their calculation, the number of infected cells/well were plotted (y) as a function of the dilution factor (x). The slope (a) and the intersection (b) with the y axis were calculated using the linear portion of the neutralization curve. Ymax was defined as the mean of infectious units at the plateau. The neutralization titer was calculated as [(Ymax/2)-b]/a. Calculation of the linear regression was possible only when infectivity was reduced by more than 50% for at least two dilutions, giving a quantification threshold of 1:40. For some samples, a reduction of infectivity ≥ 50% was observed at the 1:20 dilution only, which was confirmed at lower dilutions. Therefore, plasma samples exhibiting a reduction of infectivity ≥ 50% at the 1:20 dilution only were arbitrarily defined as having a neutralization titer of 1:20. Plasma samples exhibiting a reduction of infectivity < 50% at the 1:20 dilution were considered non-neutralizing. The value corresponding to half the detection threshold (1:10) was used when a quantitative expression of the results was necessary (on graphs and for statistical assays).

### Determination of SFV genome and coinfection

Study subjects were infected by SFV strains belonging to two genotypes (GI and GII), previously determined by direct sequencing of the *env* gene amplified from the buffy coat [33]. Here, *env* genotype-specific primers were used to detect coinfection by strains from the two genotypes. *Env* fragments were amplified from buffy coat genomic DNA using specific primers (S5 Table). Nested PCR was performed by mixing 500 ng genomic DNA in the enzyme buffer with the external primers (0.25 μM each), MgCl_2_ (3.5 mM), deoxynucleoside triphosphates (dNTPs, 200 μM each), and 0.5 μl HotStar-Taq polymerase (Qiagen) in a final volume of 50 μl. External PCR consisted of a 15-min-long denaturation step at 95°C, followed by 40 amplification cycles (45 s at 95°C, 45 s at 50°C, and 1 min per kb at 72°C) and a 7-min-long extension step at 72°C. The product (5 μl) was then used as the template for a second internal PCR under the same conditions but using the internal primers.

### Foamy vectors expressing gorilla SFV env

The expression-optimized four-component PFV vector system comprised constructs expressing PFV Gag (pcoPG4), Env (pcoPE), Pol (pcoPP), and the EGFP-expressing transfer vector puc2MD9 [76, 77]. Expression-optimized gorilla SFV envelope (*env*) genes were synthesized by Genscript (Piscataway, NJ, USA) using sequences from the GI-D468 *env* and GII-K74 *gp80*^*SU*^ genes. Restriction sites were inserted in both sequences, allowing the generation of chimeric genes through cloning (sequences are available upon request). Gorilla SFV *env* open reading frames (ORFs) were cloned into the pcoPE plasmid using the HindIII-ApaI restriction sites, replacing the PFV *env* gene. Foamy vector particles were produced by co-transfection of four plasmids. Polyethyleneimine (45 μl JetPEI, Polyplus # 101-10N, Ozyme, Montigny-le-Bretonneux, France) and 15 μg total DNA (gag:env:pol:transgene ratio of 8:2:3:32) were added to a 10-cm^2^ culture dish seeded with 4 x 10^6^ HEK 293T cells. Supernatant was collected 48 h later, clarified by centrifugation at 1,500 x g for 10 min, and stored at -80°C as single-use aliquots. Titration was performed on GFAB cells and an S6 Ultimate UV Image analyzer (CTL Europe, Bonn, Germany) was used to count fluorescently stained cells. One infectious unit was defined as a fluorescent cell, resulting from EGFP expression, for which the gene is encoded by the puc2MD9 plasmid packaged into vector particles.

### Statistics

We studied associations between categorical and continuous variables with Fisher’s exact test and Spearman’s rank test, respectively. Analyses were conducted using GraphPad Prism v5.0 (GraphPad Software, San Diego California USA). *P* values < 0.05 defined statistical significance.

## Supporting information

S1 Fig Foamy virus vectors and replicating viruses have the same susceptibility to neutralization by plasma samples from infected individuals. Viruses and vectors were incubated at the same moi (100 IU/well) with serial dilutions of plasma samples before titration on GFAB cells. Infected cells were visualized through β-galactosidase activity or fluorescence, respectively. Results are expressed as the percentage relative infectivity using untreated vector/virus as reference. Titers are indicated in the legends and the dashed lines represent the neutralization detection threshold (1:20). A: PFV virus/vector treated with plasma from individual BAD463; B: PFV virus/vector treated with plasma from individual BAD468; C: GI-D468 virus/vector treated with plasma from individual BAD463; D: GI-D468 virus/vector treated with plasma from individual BAD468; E: GII-K74 virus/vector treated with plasma from individual BAK55; F: GII-K74 virus/vector treated with plasma from individual BAK232. Neutralization titers from 13 plasma samples were determined against replicating viruses and vectors in independent experiments. G: Neutralization titers against GI vector are presented as a function of those against GI virus; H: Neutralization titers against GII vector are presented as a function of those against GII virus; correlation coefficients and *P* values from the Spearman rank test are indicated on the graphs.
(DOCX)

S2 Fig Alignments of GI and GII partial envelope protein sequences show similar variability across each genotype. The protein sequences corresponding to *env* fragments amplified by genotype-specific PCR were obtained by direct sequencing (A: Gor I genotype, aa 298–362; B: Gor II genotype, aa 286–356). The sequences of viruses used in the neutralization assay are shown at the top. The sequences of the strains are classified by neutralization titer of the plasma of SFV-infected individuals. Identical residues are indicated with dots. Sequence logos are shown at the bottom.
(DOCX)

S1 Table Study participants. Country of origin, SFV infection status, sex, ethnic group, wound, NHP species, age and duration of infection are presented.
(DOCX)

S2 Table Genotype and neutralization specificity of plasma samples from gorilla SFV-infected hunters. Viral genotypes obtained by two methods and neutralization titers against four viral strains are presented.
(DOCX)

S3 Table Neutralization titers against foamy viral vectors. Genotype, neutralization patterns and neutralization titers against four vectors carrying wild type and chimeric envelopes are presented.
(DOCX)

S4 Table Coinfection with SFV strains from both genotype I and II in apes from Cameroon and Gabon. For 27 NHPs, viral genotype, situation, place of living and reference for previous description are presented.
(DOCX)

S5 Table PCR primers used to amplify genotype-specific *env* sequences from gorilla and chimpanzee SFV. Primers and PCR parameters are described for each genotype-specific PCR.
(DOCX)
