# Supplementary material for: Potent neutralizing antibodies in humans infected with zoonotic simian foamy viruses target conserved epitopes located in the dimorphic domain of the surface envelope protein
Source: PLoS Pathog. 2018 Oct 8;14(10):e1007293. doi: 10.1371/journal.ppat.1007293 (PMC6193739; doi:10.1371/journal.ppat.1007293)
Supplement: S1 Fig — Viruses and vectors were incubated at the same moi (100 IU/well) with serial dilutions of plasma samples before titration on GFAB cells. Infected cells were visualized through β-galactosidase activity or fluorescence, respectively. Results are expressed as the percentage relative infectivity using untreated vector/virus as reference. Titers are indicated in the legends and the dashed lines represent the neutralization detection threshold (1:20). A: PFV virus/vector treated with plasma from individual BAD463; B: PFV virus/vector treated with plasma from individual BAD468; C: GI-D468 virus/vector treated with plasma from individual BAD463; D: GI-D468 virus/vector treated with plasma from individual BAD468; E: GII-K74 virus/vector treated with plasma from individual BAK55; F: GII-K74 virus/vector treated with plasma from individual BAK232. Neutralization titers from 13 plasma samples were determined against replicating viruses and vectors in independent experiments. G: Neutralization titers against GI vector are presented as a function of those against GI virus; H: Neutralization titers against GII vector are presented as a function of those against GII virus; correlation coefficients and P values from the Spearman rank test are indicated on the graphs. (DOCX) [file ppat.1007293.s001.docx]

PPATHOGENS-D-18-00733-Revised

S1 Figure: Foamy virus vectors and replicating viruses have the same susceptibility to neutralization by plasma samples from infected individuals.

Viruses and vectors were incubated at the same moi (100 IU/well) with serial dilutions of plasma samples before titration on GFAB cells. Infected cells were visualized through β-galactosidase activity or fluorescence, respectively. Results are expressed as the percentage relative infectivity using untreated vector/virus as reference. Titers are indicated in the legends and the dashed lines represent the neutralization detection threshold (1:20). A: PFV virus/vector treated with plasma from individual BAD463; B: PFV virus/vector treated with plasma from individual BAD468; C: GI-D468 virus/vector treated with plasma from individual BAD463; D: GI-D468 virus/vector treated with plasma from individual BAD468; E: GII-K74 virus/vector treated with plasma from individual BAK55; F: GII-K74 virus/vector treated with plasma from individual BAK232. Neutralization titers from 13 plasma samples were determined against replicating viruses and vectors in independent experiments. G: Neutralization titers against GI vector are presented as a function of those against GI virus; H: Neutralization titers against GII vector are presented as a function of those against GII virus; correlation coefficients and *P* values from the Spearman rank test are indicated on the graphs.
