# Supplementary material for: Potent neutralizing antibodies in humans infected with zoonotic simian foamy viruses target conserved epitopes located in the dimorphic domain of the surface envelope protein
Source: PLoS Pathog. 2018 Oct 8;14(10):e1007293. doi: 10.1371/journal.ppat.1007293 (PMC6193739; doi:10.1371/journal.ppat.1007293)
Supplement: S2 Fig — The protein sequences corresponding to env fragments amplified by genotype-specific PCR were obtained by direct sequencing (A: Gor I genotype, aa 298–362; B: Gor II genotype, aa 286–356). The sequences of viruses used in the neutralization assay are shown at the top. The sequences of the strains are classified by neutralization titer of the plasma of SFV-infected individuals. Identical residues are indicated with dots. Sequence logos are shown at the bottom. (DOCX) [file ppat.1007293.s002.docx]

PPATHOGENS-D-18-00733-Revised


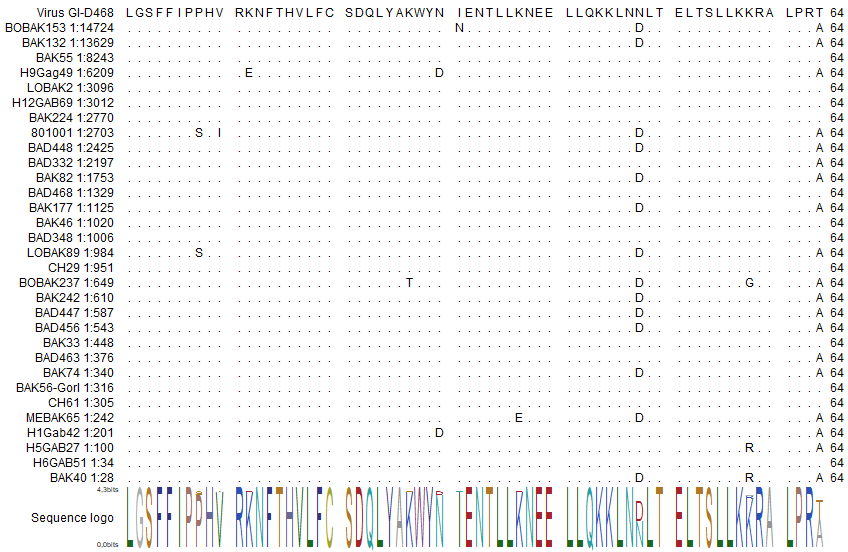


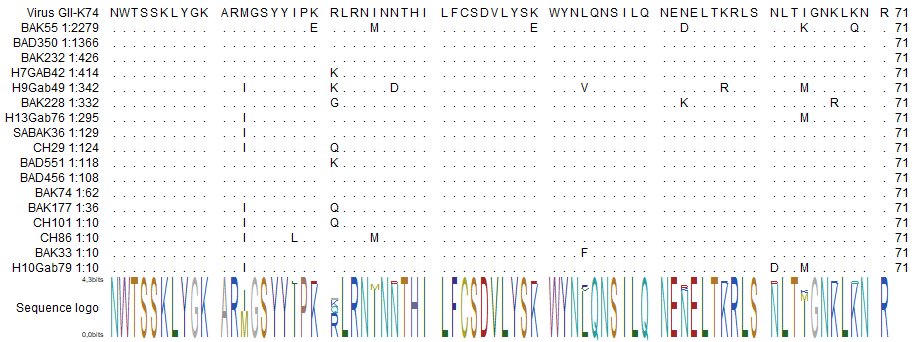


S2 Figure. Alignments of GI and GII partial envelope protein sequences show similar variability across each genotype.

The protein sequences corresponding to *env* fragments amplified by genotype-specific PCR were obtained by direct sequencing (A: Gor I genotype, aa 298-362; B: Gor II genotype, aa 286-356). The sequences of viruses used in the neutralization assay are shown at the top. The sequences of the strains are classified by neutralization titer of the plasma of SFV-infected individuals. Identical residues are indicated with dots. Sequence logos are shown at the bottom.
