# Supplementary material for: Potent neutralizing antibodies in humans infected with zoonotic simian foamy viruses target conserved epitopes located in the dimorphic domain of the surface envelope protein
Source: PLoS Pathog. 2018 Oct 8;14(10):e1007293. doi: 10.1371/journal.ppat.1007293 (PMC6193739; doi:10.1371/journal.ppat.1007293)
Supplement: S1 Table — Country of origin, SFV infection status, sex, ethnic group, wound, NHP species, age and duration of infection are presented. (DOCX) [file ppat.1007293.s003.docx]

PPATHOGENS-D-18-00733

Table S1. Study participants

| Code | Sex | Ethnicity | Wound | Animal | Age (Yrs) | Duration of infection (Yrs) ^a^ |
| --- | --- | --- | --- | --- | --- | --- |
| Cameroon ^b^, Uninfected |  |  |  |  |  |  |
| AKO255 | M | Bantu | No wound | -- | 43 | -- |
| AKO318 | M | Bantu | bite | Cercopithecus | 45 | -- |
| BAD146 | M | Bantu | No wound | -- | 59 | -- |
| BAD180 | M | Bantu | No wound | -- | 60 | -- |
| BAD19 | M | Bantu | bite | Gorilla | 44 | -- |
| BAD32 | M | Bantu | bite | Missing | 40 | -- |
| BAK165 | M | Pygmy | bite | Gorilla | 58 | -- |
| BAK231 | M | Pygmy | bite | Cercopithecus | 50 | -- |
| Cameroon, SFV-infected |  |  |  |  |  |  |
| BAD316 | M | Bantu | bite | Chimpanzee | 51 | 15 |
| BAD327 | M | Bantu | bite | Chimpanzee | 33 | 3 |
| BAD332 | M | Bantu | bite | Gorilla | 37 | 12 |
| BAD348 | M | Bantu | bite | Gorilla | 31 | 12 |
| BAD350 | M | Bantu | bite | Gorilla | 68 | 28 |
| BAD436 | M | Bantu | bite | Cercopithecus | 56 | 21 |
| BAD447 | M | Bantu | bite | Gorilla | 59 | 19 |
| BAD448 | M | Bantu | bite | Gorilla | 53 | 9 |
| BAD456 | M | Bantu | bite | Gorilla | 33 | 11 |
| BAD463 | M | Bantu | bite | Gorilla | 44 | 7 |
| BAD468 | M | Bantu | bite | Gorilla | 35 | 12 |
| BAD50 | M | Bantu | bite | Cercopithecus | 60 | 4 |
| BAD551 | M | Bantu | bite | Gorilla | 41 | 14 |
| BAK132 | M | Pygmy | bite | Gorilla | 60 | 30 |
| BAK133 | M | Pygmy | bite | Gorilla | 50 | 20 |
| BAK177 | M | Pygmy | bite | Gorilla | 39 | 13 |
| BAK188 | M | Pygmy | bite | Cercopithecus | 47 | 32 |
| BAK224 | M | Pygmy | bite | Gorilla | 38 | 19 |
| BAK228 | M | Pygmy | bite | Gorilla | 70 | 40 |
| BAK232 | M | Pygmy | bite | Gorilla | 59 | 19 |
| BAK235 | M | Pygmy | bite | Gorilla | 55 | 28 |
| BAK242 | M | Pygmy | bite | Gorilla | 49 | 19 |
| BAK33 | M | Pygmy | bite | Gorilla | 45 | 21 |
| BAK40 | M | Pygmy | bite | Gorilla | 35 | 5 |
| BAK46 | M | Pygmy | bite | Gorilla | 50 | 24 |
| BAK55 | M | Pygmy | bite | Gorilla | 68 | 38 |
| BAK56 | M | Pygmy | bite | Gorilla | 63 | 25 |
| BAK74 | M | Pygmy | bite | Gorilla | 47 | 21 |
| BAK82 | M | Pygmy | bite | Gorilla | 52 | 6 |
| BOBAK153 | M | Pygmy | bite | Gorilla | 68 | 15 |
| BOBAK237 | M | Pygmy | No wound | Gorilla | 68 |  |
| CAMVAE3 | M | Bantu | bite | Cercopithecus | 29 | 4 |
| LOBAK2 | M | Pygmy | bite | Gorilla | 78 | 48 |
| LOBAK89 | M | Pygmy | bite | Gorilla | 51 | 31 |
| MEBAK65 | M | Pygmy | bite | Gorilla | 40 | 20 |
| PYL106 | M | Pygmy | bite | Chimpanzee | 60 | 45 |
| PYL149 | M | Pygmy | bite | Chimpanzee | 60 | 15 |
| SABAK36 | M | Pygmy | bite | Gorilla | 68 | 28 |
| 801001 | M | Pygmy | bite | Gorilla | 60 | 25 |
| AG15 | M | Bantu | bite | Chimpanzee | 71 | 43 |
| AG16 | M | Bantu | bite | Cercopithecus | 43 | 20 |
| CH101 | M | Bantu | bite | Gorilla | 76 | 11 |
| CH29 | M | Bantu | bite | Gorilla | 50 | 1 |
| CH61 | M | Bantu | bite | Gorilla | 65 | 13 |
| CH65 | M | Pygmy | bite | Gorilla | 58 | 32 |
| CH66 | M | Pygmy | bite | Chimpanzee | 60 | 4 |
| CH86 | M | Bantu | bite | Gorilla | 62 | 15 |
| Gabon, SFV- infected |  |  |  |  |  |  |
| H10GAB79 | M | Bantu | bite | Gorilla | 28 | 2 |
| H12GAB69 | M | Bantu | bite | Gorilla | 38 | 16 |
| H13GAB76 | M | Bantu | bite | Gorilla | 31 | 6 |
| H14GAB34 | M | Bantu | bite | Cercopithecus | 73 | 40 |
| H1GAB42 | M | Bantu | bite | Gorilla | 65 | 12 |
| H3GAB56 | M | Bantu | bite | Chimpanzee | 48 | 1 |
| H4GAB59 | M | Bantu | bite | Chimpanzee | 51 | 1 |
| H5GAB27 | M | Bantu | bite | Gorilla | 80 | 27 |
| H6GAB51 | M | Bantu | bite | Gorilla | 56 | 28 |
| H7GAB42 | M | Bantu | bite | Gorilla | 65 | 45 |
| H9GAB49 | M | Bantu | bite | Gorilla | 58 | 25 |

Table S1. Study participants. Country of origin, SFV infection status, sex, ethnic group, wound, NHP species, age and duration of infection are presented.

^a^For SFV-infected participants, the time elapsed between receiving the wound and sampling was considered to be the duration of SFV infection; ^b^described in [1-3].

References

1. Calattini S, Betsem EBA, Froment A, Mauclere P, Tortevoye P, Schmitt C, et al. Simian foamy virus transmission from apes to humans, rural Cameroon. Emerg Infect Dis. 2007;13(9):1314-20. doi: 10.3201/eid1309.061162.

2. Betsem E, Rua R, Tortevoye P, Froment A, Gessain A. Frequent and recent human acquisition of simian foamy viruses through apes' bites in Central Africa. PLoS Pathog. 2011;7(10):e1002306. doi: 10.1371/journal.ppat.1002306.

3. Mouinga-Ondeme A, Caron M, Nkoghe D, Telfer P, Marx P, Saib A, et al. Cross-species transmission of simian foamy virus to humans in rural Gabon, Central Africa. J Virol. 2012;86(2):1255-60. doi: 10.1128/JVI.06016-11.
