# Supplementary material for: Potent neutralizing antibodies in humans infected with zoonotic simian foamy viruses target conserved epitopes located in the dimorphic domain of the surface envelope protein
Source: PLoS Pathog. 2018 Oct 8;14(10):e1007293. doi: 10.1371/journal.ppat.1007293 (PMC6193739; doi:10.1371/journal.ppat.1007293)
Supplement: S2 Table — Viral genotypes obtained by two methods and neutralization titers against four viral strains are presented. (DOCX) [file ppat.1007293.s004.docx]

PPATHOGENS-D-18-00733-Revised

Table S2: Genotype and neutralization specificity of plasma samples from gorilla SFV-infected hunters.

|  | Genotype | | Neutralization (1:titer) | | | |
| --- | --- | --- | --- | --- | --- | --- |
| Code | *env* PCR^a^ | GI and GII-specific PCR^b^ | GI-D468 | GII-K74 | CI-PFV | CII-SFV7 |
| 801001 | GI | ND | 2703 | 72 | 299 | 43 |
| BAD332 | GI | GI | 2197 | 10 | 462 | 10 |
| BAD348 | GI | GI | 1006 | 29 | 417 | 23 |
| BAD350 | GII | GII | 22 | 1366 | 10 | 2427 |
| BAD447 | GI | GI | 587 | 529 | 444 | 588 |
| BAD448 | ND | GI | 2425 | 10 | 683 | 10 |
| BAD456 | GI | GI+GII | 543 | 108 | 59 | 219 |
| BAD463 | GI | GI | 376 | 10 | 110 | 10 |
| BAD468 | GI | GI | 1329 | 59 | 299 | 66 |
| BAD551 | GII | GII | 10 | 118 | 10 | 53 |
| BAK132 | GI | GI | 13629 | 10 | 825 | 10 |
| BAK133 | ND | ND | 22 | 1264 | 10 | 8793 |
| BAK177 | GI | GI+GII | 1125 | 36 | 36 | 65 |
| BAK224 | GI | GI | 2770 | 10 | 520 | 10 |
| BAK228 | GII | GII | 10 | 332 | 10 | 488 |
| BAK232 | GII | GII | 10 | 426 | 10 | 1533 |
| BAK235 | ND | ND | 10 | 10 | 10 | 10 |
| BAK242 | GI | GI | 610 | 10 | 83 | 10 |
| BAK33 | GI | GI+GII | 448 | 10 | 22 | 10 |
| BAK40 | ND | GI | 28 | 10 | 10 | 10 |
| BAK46 | GI | GI | 1020 | 10 | 205 | 10 |
| BAK55 | GI | GI+GII | 8243 | 2279 | 1119 | 1415 |
| BAK56 | GI | GI | 316 | 10 | 74 | 10 |
| BAK74 | GII | GI+GII | 340 | 62 | 79 | 10 |
| BAK82 | GI | GI | 1753 | 10 | 253 | 10 |
| BOBAK153 | GI | GI | 14724 | 57 | 2147 | 20 |
| BOBAK237 | GI | GI | 649 | 10 | 406 | 10 |
| CH101 | GII | GII | 10 | 10 | 10 | 10 |
| CH29 | GI | GI+GII | 951 | 124 | 240 | 242 |
| CH61 | GI | GI | 305 | 65 | 52 | 34 |
| CH65 | ND | ND | 2875 | 10 | 926 | 10 |
| CH86 | ND | GII | 10 | 10 | 10 | 10 |
| H10GAB79 | ND | GII | 10 | 10 | 10 | 10 |
| H12GAB69 | GI | GI | 3012 | 381 | 164 | 312 |
| H13GAB76 | ND | GII | 10 | 295 | 10 | 362 |
| H1GAB42 | ND | GI+GII | 201 | 299 | 85 | 47 |
| H5GAB27 | GI | GI | 100 | 10 | 10 | 10 |
| H6GAB51 | GI | GI | 34 | 10 | 10 | 10 |
| H7GAB42 | GII | GII | 10 | 414 | 10 | 519 |
| H9GAB49 | ND | GI+GII | 6209 | 342 | 227 | 10 |
| LOBAK2 | GI | GI | 3096 | 10 | 1423 | 10 |
| LOBAK89 | GI | GI | 984 | 10 | 10 | 10 |
| MEBAK65 | GI | GI | 242 | 10 | 10 | 10 |
| SABAK36 | GII | GII | 10 | 129 | 10 | 304 |

Table S2: Genotype and neutralization specificity of plasma samples from gorilla SFV-infected hunters. Viral genotypes obtained by two methods and neutralization titers against four viral strains are presented.

^a^as determined by the sequence in [1], ^b^determined in this study based on the genotype-specific assay and sequence. ND: not determined.

References

1. Richard L, Rua R, Betsem E, Mouinga-Ondeme A, Kazanji M, Leroy E, et al. Cocirculation of two env molecular variants, of possible recombinant origin, in gorilla and chimpanzee simian foamy virus strains from Central Africa. J Virol. 2015;89(24):12480-91. doi: 10.1128/jvi.01798-15.
