# Supplementary material for: Potent neutralizing antibodies in humans infected with zoonotic simian foamy viruses target conserved epitopes located in the dimorphic domain of the surface envelope protein
Source: PLoS Pathog. 2018 Oct 8;14(10):e1007293. doi: 10.1371/journal.ppat.1007293 (PMC6193739; doi:10.1371/journal.ppat.1007293)
Supplement: S3 Table — Genotype, neutralization patterns and neutralization titers against four vectors carrying wild type and chimeric envelopes are presented. (DOCX) [file ppat.1007293.s005.docx]

PPATHOGENS-D-18-00733-Revised

Table S3: Neutralization titers against foamy viral vectors

|  |  | |  | Neutralisation (1:titer) | | | |
| --- | --- | --- | --- | --- | --- | --- | --- |
| Code | GI and GII-specific PCR^a^ | Neutralization pattern against replicating viruses | | EnvGI | EnvGI-  SUGII | EnvGI-SUvarGII | EnvGI-SUconGII |
| BAD348 | GI | Dual | | 1400 | 36 | 34 | 2315 |
| BAD456 | GI+GII | Dual | | 318 | 169 | 248 | 383 |
| BAD463 | GI | Single | | 1256 | 10 | 10 | 663 |
| BAD468 | GI | Dual | | 9819 | 138 | 326 | 8790 |
| BAD551 | GII | Single | | 10 | 325 | 333 | 10 |
| BAK177 | GI+GII | Dual | | 790 | 66 | 264 | 655 |
| BAK232 | GII | Single | | 10 | 733 | 406 | 10 |
| BAK33 | GI+GII | Single | | 657 | 10 | 10 | 749 |
| BAK55 | GI+GII | Dual | | 9623 | 2578 | 2290 | 5222 |
| BAK74 | GI+GII | Dual | | 1358 | 492 | 380 | 1069 |
| CH61 | GI | Dual | | 264 | 29 | 26 | 337 |
| H12GAB69 | GI | Dual | | 1931 | 222 | 325 | 1179 |
| H5GAB27 | GI | Single | | 240 | 10 | 10 | 135 |

Table S3: Neutralization titers against foamy viral vectors. Genotype, neutralization patterns and neutralization titers against four vectors carrying wild type and chimeric envelopes are presented.

^a^determined in this study based on the genotype-specific assay and sequence. ND: not determined.
