# Supplementary material for: Potent neutralizing antibodies in humans infected with zoonotic simian foamy viruses target conserved epitopes located in the dimorphic domain of the surface envelope protein
Source: PLoS Pathog. 2018 Oct 8;14(10):e1007293. doi: 10.1371/journal.ppat.1007293 (PMC6193739; doi:10.1371/journal.ppat.1007293)
Supplement: S4 Table — For 27 NHPs, viral genotype, situation, place of living and reference for previous description are presented. (DOCX) [file ppat.1007293.s006.docx]

PPATHOGENS-D-18-00733-Revised

Table S4: Coinfection with strains from both genotype I and II in apes from Cameroon and Gabon

| **NHP**^a^ | **Genotype^b^** | **Situation** | **Country** | **Described in** |
| --- | --- | --- | --- | --- |
| **Gorillas (n=8)** |  |  |  |  |
| GorGabOmo | GI | wildborn, zoo | Gabon | [1] |
| GorGabCol | GI | wildborn, zoo | Gabon | [1] |
| Tani | GI | zoo | Gabon | Unpublished |
| Djoutou | GI + GII | wildborn, zoo | Gabon | Unpublished |
| Typhen | GI + GII | wildborn, zoo | Gabon | Unpublished |
| GG04 | GI | wildborn, zoo | Gabon | Unpublished |
| GG07 | GII | wildborn, zoo | Gabon | Unpublished |
| Caroline | GI + GII | wildborn, zoo | Gabon | Unpublished |
| **Chimpanzees (n=19)** |  |  |  |  |
| PanGabNte | CI + CII | wildborn, zoo | Gabon | [2] |
| PanGabBel | CI + CII | wildborn, zoo | Gabon | [2] |
| PanGabNto | CI + CII | wildborn, zoo | Gabon | [2] |
| CpzCam35 | CI | wildborn, zoo | Cameroun | [3] |
| CpzCam15 | CI | wildborn, zoo | Cameroun | [3] |
| CpzTchWd | CI | semi-wild | Gabon | [4] |
| CpzBilWd | CI + CII | semi-wild | Gabon | [4] |
| CpzJudWd | CII | semi-wild | Gabon | [4] |
| Cpz133Wd | CII | wildborn, pet | Gabon | [4] |
| CpzMkbWd | CI | semi-wild | Gabon | [4] |
| CpzCam25 | CI | wildborn, zoo | Cameroun | Unpublished |
| CpzKdWd | CII | semi-wild | Gabon | Unpublished |
| Ayrton | CI + CII | zoo | Gabon | Unpublished |
| Junior | CII | zoo | Gabon | Unpublished |
| Brigitte | CI | wildborn, zoo | Gabon | Unpublished |
| Julie | CII | wildborn, zoo | Gabon | Unpublished |
| Gemenu | CI | wildborn, zoo | Gabon | Unpublished |
| Sindila | CI | wildborn, zoo | Gabon | Unpublished |
| Henri | CI | wildborn, zoo | Gabon | Unpublished |

Table S4: Coinfection with strains from both genotype I and II in apes from Cameroon and Gabon. For 27 NHPq, viral genotype, situation, place of living and reference for previous description are presented.

^a^Most NHPs were wild born and living in a zoo, sanctuary, or research center in Cameroon and Gabon, as described in [1-4]. ^b^We tested samples from gorillas and chimpanzees with genotype-specific PCRs.

References

1. Calattini S, Nerrienet E, Mauclere P, Georges-Courbot MC, Saib A, Gessain A. Natural simian foamy virus infection in wild-caught gorillas, mandrills and drills from Cameroon and Gabon. J Gen Virol. 2004;85:3313-7. doi: 10.1099/vir.0.80241-0.

2. Lacoste V, Mauclere P, Dubreuil G, Lewis J, Georges-Courbot MC, Gessain A. A novel gamma 2-herpesvirus of the Rhadinovirus 2 lineage in chimpanzees. Genome Res. 2001;11(9):1511-9. doi: 10.1101/gr.158601.

3. Calattini S, Nerrienet E, Mauclere P, Georges-Courbot MC, Saib A, Gessain A. Detection and molecular characterization of foamy viruses in Central African chimpanzees of the Pan troglodytes troglodytes and Pan troglodytes vellerosus subspecies. J Med Primatol. 2006;35(2):59-66. doi: 10.1111/j.1600-0684.2006.00149.x.

4. Mouinga-Ondeme A, Caron M, Nkoghe D, Telfer P, Marx P, Saib A, et al. Cross-species transmission of simian foamy virus to humans in rural Gabon, Central Africa. J Virol. 2012;86(2):1255-60. doi: 10.1128/JVI.06016-11.
