# Supplementary material for: Potent neutralizing antibodies in humans infected with zoonotic simian foamy viruses target conserved epitopes located in the dimorphic domain of the surface envelope protein
Source: PLoS Pathog. 2018 Oct 8;14(10):e1007293. doi: 10.1371/journal.ppat.1007293 (PMC6193739; doi:10.1371/journal.ppat.1007293)
Supplement: S5 Table — Primers and PCR parameters are described for each genotype-specific PCR. (DOCX) [file ppat.1007293.s007.docx]

PPATHOGENS-D-18-00733-Revised

Table S5. PCR primers used to amplify genotype-specific *env* sequences from gorilla and chimpanzee SFV

| Virus and genotype | Length (bp) | Use | Direction | Primer | Hybridization Temperature | Sequence (5’→3’) ^a^ | Site of hybridization (bp) ^b^ |
| --- | --- | --- | --- | --- | --- | --- | --- |
| GI | 588 | External PCR | Forward | SFVGIF1 | 50°C | AGGATACCATGCCGGAGTAG | 777-796 |
|  |  |  | Reverse | SFVGIR1 | 50°C | YTATTATCATCTCTTCCATCAC | 1343-1364 |
|  | 234 | Internal PCR | Forward | SFVGIF2 | 50°C | GCCCAATATGGTAATGCAAGG | 871-891 |
|  |  |  | Reverse | SFVGIR2 | 50°C | ATTCTTYCCTTGAGTGGTCC | 1085-1104 |
| GII | 506 | External PCR | Forward | SFVGIIF1 | 50°C | CCTGGACTGGCTGATGTTAG | 776-795 |
|  |  |  | Reverse | SFVGIIR1 | 50°C | TCATTRAATCGCCTATGTGG | 1261-1280 |
|  | 255 | Internal PCR | Forward | SFVGIIF2 | 50°C | ATAGAGAACATYAGACCTGC | 835-854 |
|  |  |  | Reverse | SFVGIIR2 | 50°C | TGCCCATTCGTATGGCAAGG | 1070-1089 |
| CI | 637 | External PCR | Forward | SFVCIF1 | 50°C | TTACCAGGRCATCATGCAGG | 772-791 |
|  |  |  | Reverse | SFVCIR1 | 50°C | AGGTAGATTCAGGGCTGTCC | 1389-1408 |
|  | 350 | Internal PCR | Forward | SFVCIF2 | 50°C | ATGTCCCTAARCATATGCGAC | 905-925 |
|  |  |  | Reverse | SFVCIR2 | 50°C | ACACTCGGGAACTAAATGAG | 1235-1254 |
| CII | 512 | External PCR | Forward | SFVCIIF1 | 50°C | CCTAMAGATGGCCTTATAGC | 739-758 |
|  |  |  | Reverse | SFVCIIR1 | 50°C | CAYTCAGGTACCTTATCATC | 1231-1250 |
|  | 298 | Internal PCR | Forward | SFVCIIF2 | 50°C | AGYCCTTATCCATATCAGGC | 793-812 |
|  |  |  | Reverse | SFVCIIR2 | 50°C | TAGCCCATTCAGAAGGAAGG | 1071-1090 |

Table S5. PCR primers used to amplify genotype-specific *env* sequences from gorilla and chimpanzee SFV. Primers and PCR parameters are described for each genotype-specific PCR.

^a^R = A+G, Y = C+T, M = A+C; ^b^relative to the *env* gene sequence from SFVggo_huBAD468 (GI), SFVggo_huBAK74 (GII), SFVptr_Cam15 (CI), and SFVptr_huBAD327 (CII).
